# Bronchial responses to substance P after antigen challenge in the guinea-pig: in vivo and in vitro studies

**DOI:** 10.1155/S0962935192000322

**Published:** 1992

**Authors:** E. Boichot, V. Lagente, G. Le Gall, C. Carré, J. M. Mencia-Huerta, P. Braquet, A. Lockhart, N. Frossard

**Affiliations:** 1 Laboratoire de Physiologie Respiratoire UFR Cochin-Port Royal 24 rue du Faubourg Saint Jacques Paris 75014 France; 2 Institut Henri Beaufour avenue des Tropiques Les Ulis 91952 France; 3 Bio-lnova Res. Lab. 48–52 rue de la Gare Plaisir 78370 France

## Abstract

The effect of antigen challenge on the airway responses to substance **P** and on the epithelial neutral endopeptidase (NEP) activity was investigated in aerosol sensitized guinea-pigs. In vivo, bronchial responses to aerosolized substance **P** were similar to the responses observed in antigen-challenged guinea-pigs and in the control groups. In contrast, when the guinea-pigs were pretreated with the NEP inhibitor, phosphoramidon, a significant increase in the airway responses to substance **P** was observed after antigen challenge in vivo. However, in vitro, the contractile responses of the tracheal smooth muscle to substance **P** were similar between groups of guinea-pigs, in respect to the presence or absence of the epithelium and/or phosphoramidon. Histological studies showed an accumulation of eosinophils in the tracheal submucosa after antigen challenge and intact epithelial cells. These results show that in vivo bronchial hyperresponsiveness to substance **P** after antigen challenge in the guinea-pig is not associated with increased responses of the smooth muscle to exogenous **SP** in vitro. In addition, the results with phosphoramidon suggest that loss of NEP activity cannot account for the in vivo bronchial hyperresponsiveness to substance **P** presently observed.


Research Paper

Mediators of Inflammation, 1, 207–212 (1992)

### Bronchial responses to substance P after antigen challenge in the guinea-pig: *in vivo* and *in vitro* studies

E. Boichot,*[,2] V. Lagente,[2] G. Le Gall,[1]

C. Carré,[2] J. M. Mencia-Huerta,[2]

P. Braquet,[2] A. Lockhart[1] and N. Frossard[1,CA]

[1]Laboratoire de Physiologie Respiratoire, UFR Cochin-Port Royal, 24 rue du Faubourg Saint Jacques, 75014 Paris, France;
[2]Institut Henri Beaufour, 1 avenue des Tropiques, 91952 Les Ulis, France;
* *Present Address*: Bio-Inova Res. Lab., 48–52 rue de la Gare, 78370 Plaisir, France

[CA] Corresponding Author

**THE effect of antigen challenge on the airway responses to substance P and on the epithelial neutral endopeptidase (NEP) activity was investigated in aerosol sensitized guinea-pigs. *In vivo*, bronchial responses to aerosolized substance P were similar to the responses observed in antigen-challenged guinea-pigs and in the control groups. In contrast, when the guinea-pigs were pretreated with the NEP inhibitor, phosphoramidon, a significant increase in the airway responses to substance P was observed after antigen challenge *in vivo*. However, *in vitro*, the contractile responses of the tracheal smooth muscle to substance P were similar between groups of guinea-pigs, in respect to the presence or absence of the epithelium and/or phosphoramidon. Histological studies showed an accumulation of eosinophils in the tracheal submucosa after antigen challenge and intact epithelial cells. These results show that *in vivo* bronchial hyperresponsiveness to substance P after antigen challenge in the guinea-pig is not associated with increased responses of the smooth muscle to exogenous SP *in vitro*. In addition, the results with phosphoramidon suggest that loss of NEP activity cannot account for the *in vivo* bronchial hyperresponsiveness to substance P presently observed.**

**Key words:** Aerosol, Bronchial hyperresponsiveness, Epithelium, Guinea-pig, Neutral endopeptidase, Ovalbumin, Phosphoramidon, Substance P, Trachea

### Introduction

Non-specific increase in airway responsiveness to bronchoconstrictor stimuli, namely bronchial hyperresponsiveness, is generally associated with airway inflammation in asthma. In the airways, substance P and related neurokinins, mediators of the sensory pathway, may be locally released after stimulation of C fibre endings.[1] These neuropeptides induce contraction of the airways in the guinea-pig and in human.[2,3] They also have pro-inflammatory effects in the airways[4,5] and may thus play a role in airway hyperresponsiveness.

These neuropeptides may be degraded by neutral endopeptidase (NEP), an enzyme localized to airway epithelium.[6] Blockade of NEP by phosphoramidon induces increased airway responses to exogenous and endogenous neuropeptides.[7–10] Bronchial hyperresponsiveness to substance P has been reported after viral infection or cigarette smoke.[11,12] Since the bronchoconstrictor responses to substance P were not increased after NEP blockade as compared to controls, hyperresponsiveness was attributed to an alteration in NEP activity.[11,12]

We recently developed an experimental preparation of bronchial hyperresponsiveness in which guinea-pigs are sensitized and challenged with the antigen by aerosol.[13] Enhanced bronchopulmonary responses to aerosolized and intravenous acetylcholine and 5-hydroxytryptamine are observed 24–72 h after antigen challenge associated with marked airway infiltration with eosinophils.[13] Our purpose was first to examine the bronchial responses to exogenous substance P both *in vivo* and *in vitro* in sensitized and challenged guinea-pigs. We secondly studied the influence of NEP blockade by phosphoramidon and additionally the effect of epithelium removal *in vitro* in tracheal preparations.

### Materials and Methods

Specific pathogen-free male Hartley guinea-pigs (350–400 g) (Charles River, Saint-Aubin-les-Elbeuf, France) were separated into three groups and studied at random: Group A served as control and inhaled saline aerosol twice; Group B was sensitized to ovalbumin (OA) by aerosol and challenged with saline aerosol; Group C was sensitized to and challenged with OA aerosol.

*Sensitization and challenge procedures:* Series of ten guinea-pigs were exposed for 30 min to an aerosol of OA (2 mg/ml in saline, Ultra-Neb 99, DeVilbiss, Sommerset, PA, USA) in a plexiglass chamber (30 × 50 × 30 cm) twice at 48 h intervals, as previously described.[13] 15–20 days later, guinea-pigs were challenged in the plexiglass chamber by

© 1992 Rapid Communications of Oxford Ltd exposure to aerosols of five successive increasing concentrations of OA (0.01, 0.1, 1, 5 and 10 mg/ml) for 15 min each.

*Assessment of airway responsiveness* in vivo: Airway responses to acetylcholine, histamine or substance P were assessed 48 h after challenge with antigen or saline. Guinea-pigs were anaesthetized (urethane, 1.2 g/kg, i.p.). The trachea was canulated and the lungs were mechanically ventilated with a constant tidal volume (1 ml/100 g body weight) with a respiratory pump (Ugo Basile, Varese, Italy, 60 breaths/min). Spontaneous breathing was abolished with pancuronium bromide (2 mg/kg, i.v.). Both cervical vagi nerves were transsected at the level of the neck. Pulmonary inflation pressure (PIP, mmHg) was measured as an index of the intrathoracic airway calibre by a pressure transducer (Gould PE10, Cleveland, OH, USA) connected to a lateral port of the ventilation circuit. After a 10 min equilibration period, a single 1 min aerosol administration of substance P (0.1 or 1 mg/ml) or four successive 1 min aerosol administration at 10 min interval of acetylcholine (50, 100, 200, 500 $\mu$g/ml) or histamine (1, 3, 10, 30 $\mu$g/ml) were performed. The time course of the bronchial response to substance P was studied for 10 min. The aerosol was generated by an ultrasonic nebulizer ("Pulmosonic" DeVilbiss), permanently connected in series with the afferent limb of the ventilator circuit.[13]

Two different phosphoramidon pretreatments were used: (i) conscious guinea-pigs were exposed for 15 min to an aerosol of phosphoramidon (0.1 mM in saline), 15 to 60 min before substance P (0.1 mg/ml); (ii) phosphoramidon (1 mg/kg) was administered intravenously to anaesthetized guinea-pigs through the jugular vein 5 min before substance P (0.1 mg/ml).

*Organ bath studies:* In separate experiments, guinea-pigs were killed by cervical dislocation. The trachea was dissected out, carefully cleaned of connective tissue and immersed in Krebs' solution bubbled with 95% $O_2$–5% $CO_2$. The trachea was opened longitudinally through the cartilage and carefully cut into eight strips of four cartilaginous ring segments. De-epithelialization of some ring segments was achieved by gently rubbing the luminal surface with a cotton wool swab.[14] Tracheal strips were suspended in 10 ml organ baths containing Krebs' solution bubbled with 95% $O_2$–5% $CO_2$, pH 7.4, at 37°C. The tissue was washed three times every 10 min under an initial 0.8 g tension and equilibrated to a final 2 g basal tension.[14] When the enzyme inhibitor phosphoramidon (10 $\mu$M) was used, a 30 min preincubation with the tissue was performed before substance P addition in a cumulative fashion (0.01 nM–10 $\mu$M). For SP experiments, three groups of animals were studied. For clarity in Figure 3, results are presented for the two groups of sensitized guinea-pigs (groups B and C), since unsensitized animals (group A) had a similar response to group B. Changes in smooth muscle tension, expressed in mg of contraction, were measured isometrically using Narco F60 force-displacement transducers and recorded on MKIV physiographs (Narco, Houston, TX, USA).

*Histology:* In separate experiments, tracheas from anaesthetized guinea-pigs were dissected out 48 h after antigen or saline exposure and fixed in a buffered 10% formalin solution, pH 7.4, embedded in paraffin. 5 $\mu$m sections were stained either with haematoxylin-eosin or with Luna's reagent specific for eosinophil granule content.[15] Light microscopy was carried out with a Leitz Aristoplan microscope (Rueil-Malmaison, France) and a 64 × magnification. For each guinea-pig, four to five tracheal sections were chosen at random and eosinophils were counted in a blind fashion.

*Materials:* Ovalbumin (OA, chicken egg, grade V), acetylcholine chloride, histamine dihydrochloride, phosphoramidon [N-($\alpha$-rhamno-pyranosyloxy-hydroxyphosphinyl)-L-leucyl-L-tryptophan, $NH_4$ salt] were from Sigma (St Louis, MO, USA). The other drugs used were urethane (ethylcarbamate, Prolabo, Paris, France), pancuronium bromide (Pavulon®, Organon, Fresnes, France), substance P (Novabiochem, Cléry en Vexin, France). Dilutions of all drugs were done extemporaneously in saline (NaCl, 0.9%) (*in vivo* experiments) or in modified Krebs' buffer with the following composition (mM): NaCl, 120; KCl, 5.9; $CaCl_2$, 2.5; $MgSO_4$, 1.2; glucose, 5.6; $KH_2PO_4$, 1.2; and $NaHCO_3$, 25.5 (*in vitro* experiments).

*Expression of the results and statistical analysis:* Results are expressed as means ± SEM. Statistical differences of the dose—response curves (*in vivo* and *in vitro*) or of the time-courses of the response to substance P *in vivo* between the three groups of guinea-pigs were analysed by a two-way analysis of variance. In *in vitro* experiments, $EC_{50}$ values for acetylcholine and histamine have been compared by the unpaired *t*-test.

### Results

*Effect of OA challenge on airway responses to acetylcholine and histamine: In vivo* no significant difference in the initial pulmonary inflation pressure (PIP) was observed before stimulation with either acetylcholine, histamine or substance P between the different groups of guinea-pigs. Increasing doses of acetylcholine induced a dose-related increase in PIP (Figure 1). The responses observed in non-sensitized or

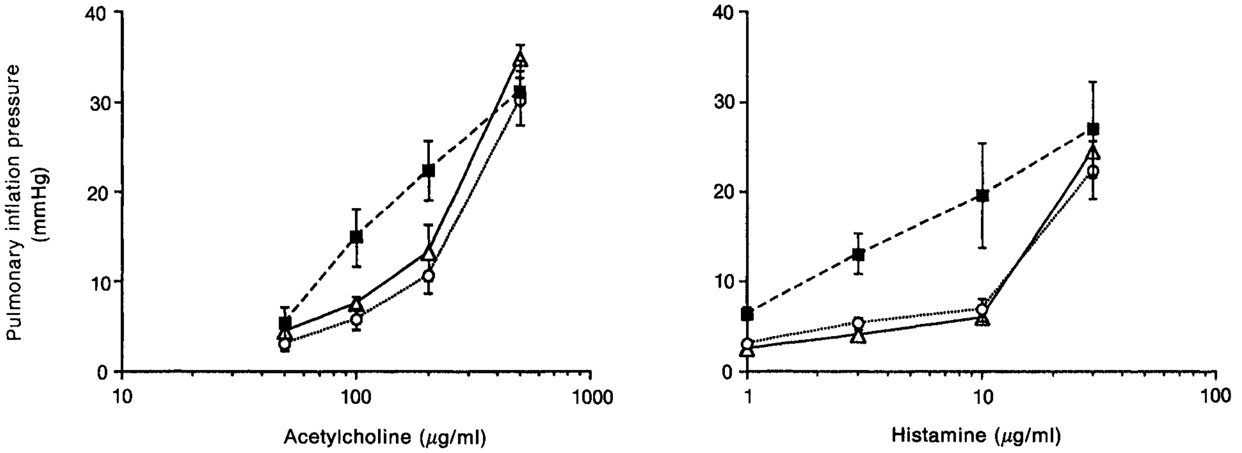

FIG. 1. Bronchopulmonary responses to four successive doses of acetylcholine (*left panel*) or histamine (*right panel*) administered by aerosol for 1 min to anaesthetized guinea-pigs from groups A, non-sensitized saline-exposed (△); B, ovalbumin sensitized saline exposed (○); and C, ovalbumin sensitized, ovalbumin exposed (■) guinea-pigs. Values are means ± SEM (bars) of five to six experiments. Significant increases in PIP were observed in group C as compared to groups A and B for both acetylcholine and histamine ($p < 0.01$).

sensitized guinea-pigs challenged with saline, (groups A and B) were similar. By contrast, after OA challenge in sensitized guinea-pigs (group C), the dose—response curve to acetylcholine was significantly shifted to the left ($p < 0.01$). Similarly, increasing doses of histamine induced a greater increase in PIP after OA challenge (group C) as compared to control groups (A and B) ($p < 0.01$) (Figure 1).

*In vitro* in the presence of tracheal epithelium, acetylcholine concentration—response curves were similar for the three groups of guinea-pigs (Table 1). The curves did not reach a plateau even at 10 mM acetylcholine. When the epithelium was removed, the plateau of contraction was observed at 3 mM acetylcholine in all groups. Although maximal contraction was slightly lower than in the presence of epithelium, $EC_{50}$ values decreased significantly (Table 1).

Histamine concentration—response curves also were not modified by OA sensitization and/or challenge either in the presence or absence of airway epithelium (Table 1). $EC_{50}$ values decreased significantly when epithelium was removed in all three groups although maximal contraction (at 0.3 mM with and 0.1 mM without epithelium) was lower.

*Effect of OA challenge on airway responses to substance P: In vivo*, substance P (1 mg/ml) induced a small increase in PIP that did not significantly differ between groups (Figure 2). Pretreatment with phosphoramidon either by inhalation (Figure 2, middle panel) or intravenously (Figure 2, lower panel) markedly enhanced the response to substance P in the three groups of animals and 1/10 of the dose of substance P (0.1 mg/ml) was used. However, the enhancement of the response was significantly greater in OA challenged (group C) than in saline challenged animals (groups A and B) ($p < 0.01$, Figure 2).

*In vitro* in the absence of phosphoramidon, concentration-response curves to substance P were similar in sensitized guinea-pigs, whether challenged with OA (group C) or saline (group B) (Figure 3) and in non-sensitized guinea-pigs (group A, not shown). In the absence of epithelium, contractions were of higher amplitude than contractions observed in the presence of the

**Table 1.** $EC_{50}$ values (μM) and maximal contractions (mg) to acetylcholine (ACh) and histamine (Hist) in trachea of guinea-pigs from groups A (inhaled saline aerosol twice), B (sensitized to OA aerosol and challenged with saline aerosol) and C (sensitized to and challenged with OA aerosol) in the presence (+E) and absence (−E) of the epithelium. Values are means ± SEM of five to six experiments. * indicates $p < 0.05$ and ** $p < 0.02$. No difference was observed between groups in the presence or absence of epithelium, respectively

| Groups | | A | | B | | C | |
|---|---|---|---|---|---|---|---|
| | | +E | −E | +E | −E | +E | −E |
| ACh | $EC_{50}$ | 8.9 ± 5.9 | 1.5 ± 0.9* | 16.2 ± 10.3 | 1.7 ± 0.6** | 10.4 ± 7.5 | 2.9 ± 1.6* |
| | Max Cont | 2207 ± 359 | 1638 ± 200 | 2012 ± 408 | 1551 ± 320 | 2075 ± 411 | 1556 ± 380 |
| Hist | $EC_{50}$ | 7.6 ± 2.2 | 3.4 ± 1.6* | 15.1 ± 6.4 | 3.8 ± 1.9* | 12.6 ± 2.9 | 2.3 ± 0.7** |
| | Max Cont | 2025 ± 358 | 1544 ± 75 | 2268 ± 361 | 1437 ± 199 | 2377 ± 342 | 1486 ± 82 |

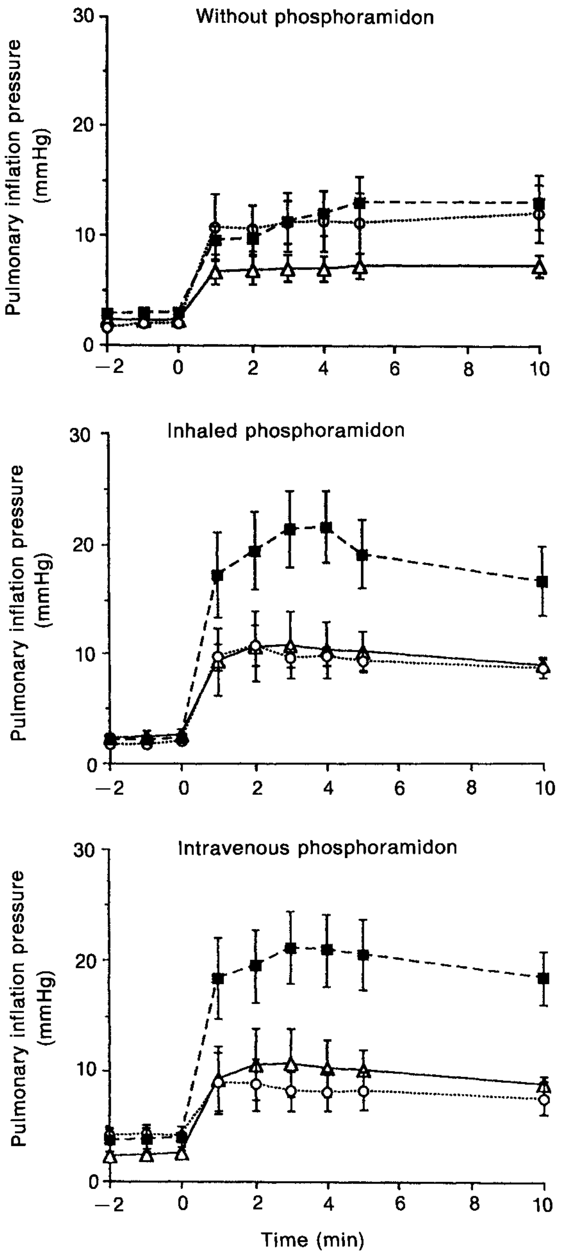

FIG. 2. Time course of the increase in pulmonary inflation pressure to inhaled substance P in guinea-pigs from groups A (1 mg/ml, △), B (0.1 mg/ml, ○) and C (0.1 mg/ml, ■): (*upper panel*) after inhalation of saline, (*middle panel*) after inhalation of phosphoramidon and (*lower panel*) after i.v. injection of phosphoramidon. Values are means ± SEM (bars) of seven to eight experiments. Significant increases in PIP were observed in group C as compared to groups A and B after pretreatment with phosphoramidon ($p < 0.01$).

epithelial layer and a significant shift to the left was observed (Figure 3). However, a plateau of contraction was not achieved at the highest concentration of substance P used (10 μM) neither in the presence nor absence of the epithelium.

Preincubation of tracheal segments with phosphoramidon (10 μM) considerably enhanced contraction of the intact trachea to substance P as reflected by an increase in the amplitude of contraction associated with a significant shift to the left of the concentration—response curves (Figure 3). The increase was similar in sensitized animals (groups B and C, Figure 3) and in non-sensitized guinea-pigs (group A, not shown). However, the plateau of contraction also was not achieved. In epithelium-free trachea, a plateau of contraction was observed at 1 μM substance P and, additionally, a significant shift to the left was observed.

*Histology:* There was a marked accumulation of eosinophils in the subepithelial and epithelial layer of tracheas from sensitized animals 48 h after OA challenge (group C) as compared with saline challenge (group B). Numbers of eosinophils in the tracheal submucosa after OA challenge (group C, $n = 5$) were 677.8 ± 55.5 per $mm^2$ compared to 76.4 ± 18.0 per $mm^2$ in saline exposed guinea-pigs (group B, $n = 4$). Nevertheless, tracheal epithelium appeared normal in guinea-pigs from both groups B and C (Figure 4).

### Discussion

The present study shows that antigen challenge in aerosol sensitized guinea-pigs induces *in vivo* airway hyperresponsiveness to substance P, acetylcholine and histamine, which is not associated with any modification of the smooth muscle response to these agents *in vitro* nor of its modulation by airway epithelium. Forty-eight hours after antigen challenge in sensitized guinea-pigs, a nonspecific bronchial hyperresponsiveness was observed *in vivo* as a leftward shift of the dose—response curves to both acetylcholine and histamine compared to that of control animals. Conversely, in sensitized guinea-pigs challenged with saline, responses were similar to that of control animals, suggesting that bronchial hyperresponsiveness was clearly due to antigen challenge, rather than to sensitization itself.

In phosphoramidon pretreated animals, exposure of sensitized guinea-pigs to antigen also markedly enhanced airway responses to substance P compared with saline challenged and control guinea-pigs. However, in sensitized animals not pretreated with phosphoramidon, no increased response to substance P was observed after antigen challenge. These observations suggest that the exogenous blockade of NEP, i.e., the prevention of substance P degradation, is necessary to visualize bronchial hyperresponsiveness to substance P. This additionally suggests that neutral endopeptidase is still functional in the airways after antigen challenge. This hypothesis is strengthened by our results obtained *in vitro*, where the responses of tracheal segments to cumulative addition of substance P were similar in the three groups of animals irrespective of the experimental conditions used:

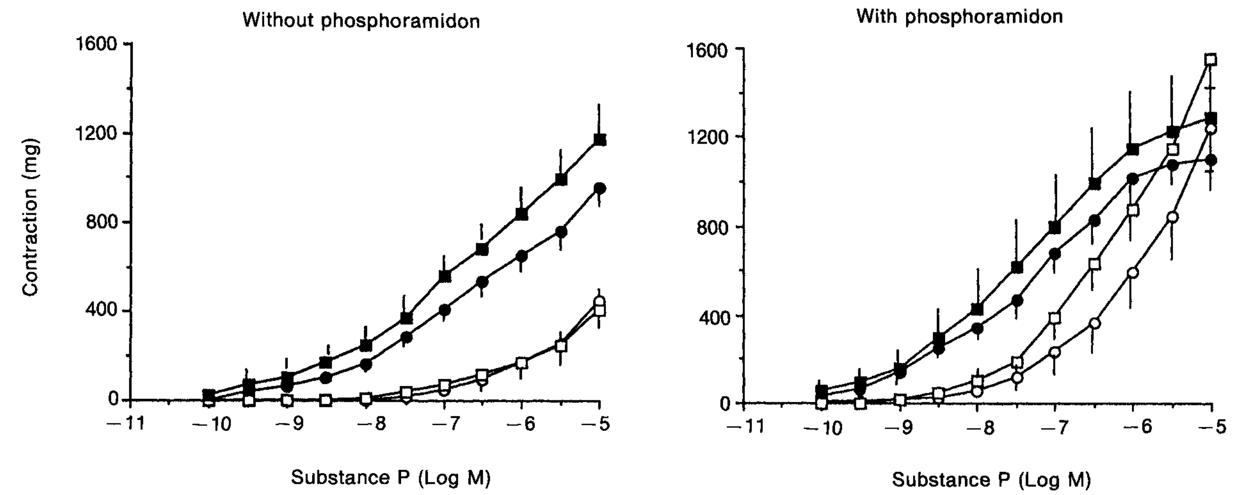

FIG. 3. Concentration–response curves to substance P on guinea-pig trachea from groups B (○, ●) and C (□, ■) in the absence and presence of phosphoramidon and in the absence (closed symbols) and presence (open symbols) of tracheal epithelium. Values are means ± SEM of five to six experiments. No difference was observed between groups B and C. However, a significant shift to the left was observed after epithelium removal in the presence or absence of phosphoramidon ($p < 0.05$). Similar curves were obtained from group A.

presence of absence of epithelium, presence or absence of the NEP inhibitor, phosphoramidon. Similarly, no difference was observed between groups in the response to acetylcholine or histamine. The very low contractile response to substance P observed in intact tracheal segments was potentiated to a similar extent by epithelium removal and by pretreatment with phosphoramidon

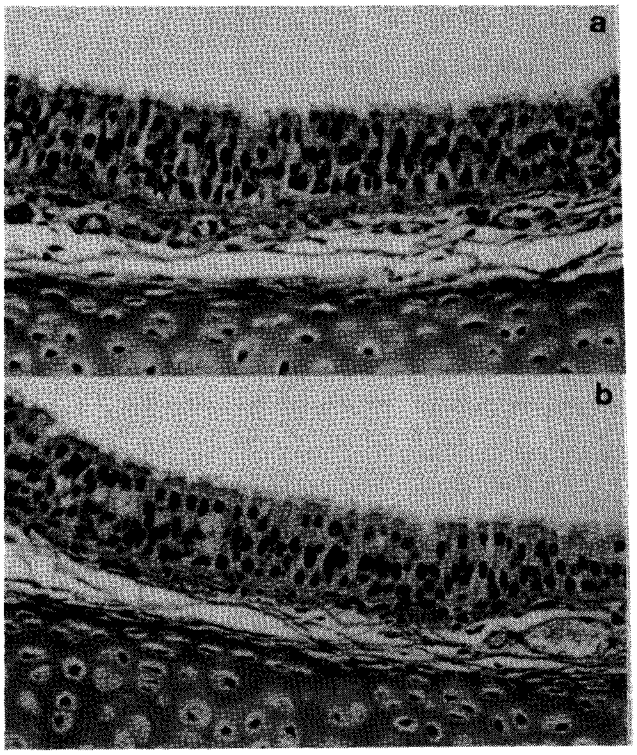

FIG. 4. Photomicrographs of tracheal sections stained with haematoxylin and eosin. Tracheas were obtained 48 h after exposure to (a) saline (group B) or (b) ovalbumin (group C) from aerosol sensitized guinea-pigs. Magnification ×64.

in the presence of epithelium. Epithelium removal and NEP blockade had a similar effect in the three groups of animals, suggesting that epithelium contains NEP degrading substance P and thus decreases airway contractility. This is in accordance with previous findings in control animals[8,14,16] and leads to the suggestion that epithelial NEP was equally active in the three groups of animals, therefore that NEP was still functional after antigen challenge.

Hence, observations that (1) bronchial hyperresponsiveness to substance P was only observed in phosphoramidon pretreated, antigen challenged animals and (2) there was no difference in the responsiveness to substance P *in vitro* after antigen challenge suggest that bronchial hyperresponsiveness exists in sensitized and challenged guinea-pigs in the absence of epithelium alteration or NEP dysfunction. It is noteworthy that both epithelium damage and loss of NEP function have been proposed as factors of bronchial hyperresponsiveness in asthmatic patients[17] in whom epithelium destruction was observed[18] and NEP inactivation was suggested by analogy with its inactivation in experimental animals after viral infection.[11,19] In our study in guinea-pigs sensitized and challenged with antigen, this absence of epithelial dysfunction is in accordance with the histological study of tracheal specimens where no obvious alterations of the epithelial layer could be noted.

The present study also shows that antigen challenge induced a marked accumulation of eosinophils in the tracheal wall, similar to that we previously reported in the lower airways and particularly in the peribronchial area and submucosa.[13] A role for eosinophils and their granule proteins has been hypothesized as a mechanism involved in bronchial hyperresponsiveness. Eosinophil proteins such as major basic protein (MBP) are toxic for guinea-pig tracheal epithelium[20] *in vitro*. In the present study, airway eosinophilia did not appear to be either directly associated with increased response of tracheal smooth muscle *in vitro* or to epithelium disruption. We may hypothesize that chronic contact with the antigen as occurs in asthmatic airways might induce more profound changes in the airways, associated with more persistent bronchial hyperresponsiveness.

Hence, it seems from our study that some factors necessary for bronchial hyperresponsiveness *in vivo* are absent *in vitro*. A step in the description of such factors would be the determination of the degree of eosinophil activation, which are infiltrated in central and more peripheral airways. However, Iijima *et al.*[21] reported that the eosinophils infiltrating the bronchioli after allergen challenge in the guinea-pig did not appear degranulated. Another explanation could be that alteration of smooth muscle mechanics and/or contractility is not the primary factor of bronchial hyperresponsiveness.

In summary, this study indicates that pathophysiological changes associated with airway hyperresponsiveness induced by antigen challenge in sensitized guinea-pigs do not involve changes in the intrinsic properties of airway smooth muscle. The presence of eosinophils within the bronchial mucosa, although associated with *in vivo* bronchial hyperresponsiveness, is not sufficient to alter morphology of epithelial cells or to induce epithelial NEP dysfunction. There are, of course, several other ways of considering airway hyperresponsiveness, such as modification of the neural influences or of the vascular responsiveness. Such findings in our experimental model may help towards a better understanding of the airway hyperresponsiveness which occurs in asthma.

### References

1. Barnes PJ. Asthma as an axon reflex. *Lancet* 1986; **1**: 242–246.
2. Lundberg JM, Saria A, Brodin E, Rosell S, Folkers K. A substance P antagonist inhibits vagally induced increase in vascular permeability and bronchial smooth muscle contraction in the guinea pig. *Proc Natl Acad Sci* 1983; **80**: 1120–1124.
3. Lundberg JM, Martling CR, Saria A. Substance P and capsaicin-induced contraction of human bronchi. *Acta Physiol Scand* 1983; **119**: 49–53.
4. McDonald DM. Neurogenic inflammation in the rat trachea. I. Changes in venules, leukocytes and epithelial cells. *J Neurocytology* 1988; **17**: 583–603.
5. Braunstein G, Fajac I, Lacronique J, Frossard N. Clinical and inflammatory responses to exogenous tachykinins in allergic rhinitis. *Am Rev Resp Dis* 1991; **144**: 630–635.
6. Johnson AR, Ashton J, Schulz WW, Erdös EG. Neutral metalloendopeptidase in human lung tissue and cultured cells. *Am Rev Respir Dis* 1985; **132**: 564–568.
7. Stimler-Gerard NP. Neutral endopeptidase-like enzyme controls the contractile activity of substance P in guinea-pig lung. *J Clin Invest* 1987; **79**: 1819–1825.
8. Sekizawa K, Tamaoki J, Graf PD, Basbaum CB, Borson DB, Nadel JA. Enkephalinase inhibitor potentiates mammalian tachykinin-induced contraction in ferret trachea. *J Pharmacol Exp Ther* 1987; **243**: 1211–1217.
9. Thompson JE, Sheppard D. Phosphoramidon potentiates the increase in lung resistance mediated by tachykinins in guinea-pigs. *Am Rev Respir Dis* 1988; **137**: 337–340.
10. Umeno E, Nadel JA, Huang HT, McDonald DM. Inhibition of neutral endopeptidase potentiates neurogenic inflammation in the rat trachea. *J Appl Physiol* 1989; **66**: 2647–2652.
11. Dusser DJ, Jacoby DB, Djokic TD, Rubinstein I, Borson DB, Nadel JA. Virus induces airway hyperresponsiveness to tachykinins: role of neutral endopeptidase. *J Appl Physiol* 1989; **67**: 1504–1511.
12. Dusser DJ, Djokic TD, Borson DB, Nadel JA. Cigarette smoke induces bronchoconstrictor hyperresponsiveness to substance P and inactivates airway neutral endopeptidase in the guinea-pig. Possible role for free radicals. *J Clin Invest* 1989; **84**: 900–906.
13. Boichot E, Lagente V, Carré C, Waltmann P, Mencia-Huerta JM, Braquet P. Bronchial hyperresponsiveness and cellular infiltration in the lung of guinea-pigs sensitized and challenged by aerosol. *Clin Exp Allergy* 1991; **21**: 67–76.
14. Frossard N, Rhoden KJ, Barnes PJ. Influence of epithelium on guinea pig airway responses to tachykinins: role of endopeptidase and cyclooxygenase. *J Pharmacol Exp Ther* 1989; **248**: 292–298.
15. Luna LG. *Manual of Histologic Staining Methods of the Armed Forces Institute of Pathology.* 3rd Ed. New York, McGraw-Hill, 1968; 258.
16. Devillier P, Advenier C, Drapeau G, Marsac J, Regoli D. Comparison of the effects of epithelium removal and of enkephalinase inhibitor on the neurokinin-induced contractions of guinea-pig isolated trachea. *Br J Pharmacol* 1988; **94**: 675–684.
17. Drazen JM, Shore SA, Gerard NP. Enzymatic degradation of neuropeptides: a possible mechanism of airway hyperresponsiveness. In: *Biochemistry of the Acute Allergic Reactions.* 5th Int. Symposium 1989; 45–56.
18. Laitinen LA, Heino M, Laitinen A, Kava T, Haahtela T. Damage of the airway epithelium and bronchial reactivity in patients with asthma. *Am Rev Respir Dis* 1985; **131**: 599–606.
19. McDonald DM. Respiratory tract infections increase susceptibility to neurogenic inflammation in the rat trachea. *Am Rev Respir Dis* 1988; **137**: 1432–1440.
20. Frigas E, Gleich GJ. The eosinophil and the pathophysiology of asthma. *J Allergy Clin Immunol* 1986; **77**: 527–537.
21. Iijima H, Ishii M, Yamauchi K, *et al.* Bronchoalveolar lavage and histological characterization of late asthmatic response in guinea-pigs. *Am Rev Respir Dis* 1987; **136**: 922–929.

ACKNOWLEDGEMENT. This work was supported by INSERM (88-5012).

**Received 27 March 1992; accepted in revised form 15 April 1992**
